# Under the radar: How participating in a student organization can shape medical students' professional identity

**DOI:** 10.1111/medu.15609

**Published:** 2025-02-10

**Authors:** Indah Puspasari Kiay Demak, Jelle Prins, Nur Meity, Tineke Bouwkamp‐Timmer, Joke Fleer, Marco Antonio de Carvalho‐Filho

**Affiliations:** ^1^ Wenckebach Institute (WIOO), Lifelong Learning, Education, and Assessment Research Network (LEARN) University Medical Center Groningen Groningen The Netherlands; ^2^ Medical Education Unit, Faculty of Medicine University of Tadulako Palu Indonesia; ^3^ Medical Education Unit, Faculty of Medicine University of Alkhairaat Palu Indonesia; ^4^ Department Health Sciences Section Health Psychology University Medical Center Groningen Groningen The Netherlands

## Abstract

**Background:**

Medical students' professional identity formation (PIF) starts early in their academic journey and is shaped by diverse social influences. Research shows that while participation in student organizations cultivates essential skills, it may also reinforce homogeneity and prevent cultural change. However, the impact of student organizations on PIF remains under‐researched. This study aimed to investigate how a particular student organization impacted the PIF of novice students.

**Methods:**

We conducted a qualitative study utilizing constructivist grounded theory and the rich pictures methodology. We interviewed 12 novices, six senior students, three alumni and seven teachers from a medical school in Indonesia. The interviews with students were facilitated by Rich Pictures. The transcripts and pictures were iteratively analysed.

**Results:**

Novice students (i.e. first‐year medical students) participated in an orientation programme organized by a centralized student organization. Becoming a member of this organization facilitated access to extracurricular training and networking. During the onboarding to this organization, senior students imparted values professed by the student organization: hierarchy, camaraderie and confidentiality. However, the way the seniors put these values into practice deviated from their intended purpose, leading to a mismatch that the novices perceived as oppressive. After an initial phase of resistance, novices entered a negotiation process to decide whether to persist with the orientation programme, resulting in three distinct outcomes: internalizing the values and being accepted as a member, enduring the programme by role‐playing or becoming an outsider. This negotiation was accompanied by intense emotional suffering and identity dissonance. This socialization process ended up reinforcing an often oppressive hierarchical culture, which prevented novices from becoming change agents.

**Conclusion:**

Participating in this student organization significantly influences PIF, and developing survivorship bias may prevent students from enacting transformative change. Reforming this often oppressive system would require collaboration among faculty, teachers, student organizations and students.

## Abstract 2

Discover how socialization processes associated with student organizations shape the professional identity of novice medical students through research from Demak et al. #MedStudentLife #ProfessionalIdentity #MedicalEducation

## 1 INTRODUCTION

Professional identity formation (PIF) is crucial for medical students as it shapes their sense of self within the medical profession and influences their values, attitudes and future behaviours as health care practitioners.^1^ Student organizations may play a pivotal role in fostering medical students' PIF by facilitating the transfer and internalization of values through providing socialization experiences that connect novices with senior members. However, as these experiences often happen under the radar of medical educators, it is possible that negative aspects of the medical culture related to the hidden curriculum in medicine, such as oppressive hierarchical practices, are reproduced and reinforced within these organizations, preventing students to become agents of change. Without understanding how student organizations influence medical students' PIF, medical educators cannot develop educational and supportive interventions to mitigate this impact and ensure healthy and nurturing socialization experiences for novice medical students.

Professional identity in medicine is defined as ‘a representation of self, achieved in stages over time during which the characteristics, values, and norms of the medical profession are internalized, resulting in an individual thinking, acting, and feeling like a physician’.^1^ PIF occurs concurrently within three distinct domains, all of which are relevant to medical education. These domains encompass individual, relational and collective identities. The individual domain focuses on personal growth and values, the relational domain emphasizes interactions with others in the medical context and the collective domain relates to a shared identity within the broader medical community.^2^, ^3^ Together, these domains contribute to the holistic development of medical professionals, influencing how they perceive themselves, interact with others and contribute to the profession.^2^, ^4^ This process of developing a professional identity happens through the socialization of medical students within the medical community. It is through their socialization within the medical community that students cultivate a collective identity by internalizing explicit and implicit norms and values. This is the arena where the individual, relational and collective identities will merge into a meaningful self.^2^, ^3^, ^5^, ^6^

Student organizations may play a crucial role in medical students' socialization.^7^ In the literature, several synonyms for student organizations are used, including ‘student association’, ‘student society’, ‘student council’, ‘student union’, ‘student club’ and ‘student government’.^7^, ^8^, ^9^, ^10^ The activities of student organizations can be formal, aligned with curricular activities, or they can be informal extracurricular activities that enhance learning and development beyond the curriculum. These activities can have a socio‐political dimension or focus on personal development and well‐being. Involvement in student organizations is thus multifaceted and may encompass academic, social and political elements.^11^, ^12^ In general, student organizations are considered miniature representations of the future professional community of practice.^13^

Participation in student organizations may yield favourable outcomes, such as enhanced communication skills, discipline, collaborative abilities, access to and analysis of information, critical thinking, problem‐solving skills and adaptability.^11^, ^14^ Student organizations are particularly effective for fostering class formation and bonding.^7^ Specifically for first‐year students, becoming involved in student organizations can be a valuable part of their transition from high school to university, since it fosters a sense of belonging and promotes reflection on their values, identity and purpose.^15^ However, it is essential to recognize that these organizations can also exhibit characteristics such as the tendency to reinforce community homogenization, perpetuate existing hierarchies and power structures and demonstrate negative role modelling and mistreatment of members.^6^, ^16^, ^17^

Considering the potential relevance of student organizations in shaping the socialization processes of medical students, we believe that shedding light on the role they play in students' professional identity is of paramount importance for medical educators committed to supporting PIF. Furthermore, it can provide valuable guidance on pedagogical approaches student organizations can adopt to facilitate optimal PIF. To explore the socialization process within student organizations, this study explores a unique context where a centralized student organization serves as an umbrella, overseeing and coordinating various other student organizations. The central question guiding this study is: How does participating in a student organization at an Indonesian medical school shape medical students' PIF?

## 2 METHODS

### 2.1 Design

In this exploratory qualitative study, under a constructionist paradigm, we adopted the constructivist grounded theory and the rich pictures methodologies. Grounded theory offers a set of guidelines and procedures that serve as a way to learn about the worlds we study and an inductive methodology for constructing theories to understand them.^18^ We used a combination of interviews and the rich pictures technique to capture the complex situation and interactions between the students, especially emotional aspects that are difficult to express in words. Rich pictures are visual depictions of specific events, persons involved, their feelings, actions and behaviours, as well as any other influences that may have been at play. This technique has been used widely in health profession education research.^19^

#### 2.1.1 Context

We conducted our study in a Medical School in Indonesia, a country that is classified as having a collectivist culture, scoring high on the power distance and low on the uncertainty avoidance dimension.^20^ In general, medical education in Indonesia involves two stages: preclinical and clinical. The preclinical stage lasts 3.5–4 years and the clinical stage 2 years. In most medical schools in Indonesia, there is a tradition for first‐year students to participate in an orientation programme, organized by the university, the faculty and the student executive board. In our medical school, there are two orientation programmes. The first is an obligatory, full‐day, 1‐week orientation programme organized by the university and the faculty that happens before the academic activities begin. The second is organized by the student executive board, or the Badan Eksekutif Mahasiswa (BEM), which is the leading, centralized student organization. BEM has several divisions and aims to facilitate personal and professional development in diverse dimensions, such as clinical competence, research, arts and religion.

One of the main aims of the BEM orientation programme is to introduce novice students not only to the community of medical students but also to the broader medical profession. Indonesian doctors are guided by several core values outlined in the physician's oath and medical code of ethics, emphasizing a commitment to solidarity with colleagues, the confidentiality of patient information and deep respect for teachers and mentors.^21^ The medical field in Indonesia also values a culture of seniority, where junior doctors are expected to learn from and respect the experience of their seniors. This hierarchical structure is intended to foster mentorship and ensure the transfer of knowledge and values from one generation to the next, thereby reinforcing professional ethics within the medical community.

### 2.2 Participants and ethical considerations

To comprehend how the BEM activities influence students' PIF, we interviewed novices, senior students, alumni and faculty members (Table 1). We used purposive sampling to include students who were involved in the BEM activities, as well as those who were not.

**TABLE 1:** Participants' characteristics.

Subject	Gender		Age	Senior students' academic year	Faculty's years of experience in the educational field
	Male	Female			
Novice students (N1–N12)	6	6	18–20	N/A	N/A
Senior students (S1–S6)	4	2	18–25	2nd–6th year	N/A
Alumni (A1–A3)	2	1	25–33	N/A	N/A
Faculty members (FM1–FM7)	‐	7	32–60	N/A	7–15 years

Abbreviation: N/A, not applicable.

We interviewed 12 novices throughout their first year. Our target groups included novices currently participating in the BEM orientation programme, those who had completed it and those who did not participate in the programme. We invited novices via a WhatsApp group and informed them that the interviews were online and required at least 2 h. Novices interested in the study sent questions via email or WhatsApp directly to the first author before deciding whether to participate.

We interviewed senior students to gain a deeper understanding of how students make sense of their socialization within the BEM. We also considered it important to explore the long‐term professional and personal consequences of becoming a member or choosing not to. We invited six senior students with different layers of interaction within the BEM, ranging from peripheral and core members to outsiders of the BEM. We also invited three former BEM core alumni to understand how the organization evolved along its history and how former members relate to BEM's policies and activities.

Still, during data analysis and collection, we understood that there is a complex interplay between the BEM and faculty members. Student participants shared conflicting perspectives on how faculty members would approve or disapprove BEM activities. These different perspectives made it clear for the research group that exploring faculty views on the BEM would add to our understanding of the mechanisms by which the BEM was influencing students' PIF. We selected seven faculty members based on their roles. We opted for faculty leaders, department heads, BEM advisers and regular faculty members to understand how the BEM influenced students' personal and professional development.

We obtained ethical approval from The Medical and Health Research Ethics Committee of the Faculty of Medicine Tadulako University in May 2022 (3346/UN28.1.30/KL/2022). We informed the participants about the purpose of the study and assured them that the interview data would be used anonymously and treated with confidentiality. All participants signed informed consent before the interview began.

### 2.3 Data collection

We collected the data between May 2022 and February 2023. Our study began by gathering data through rich pictures and interviews with novice and senior students. During the iterative analysis process, we recognized the importance of capturing a broader range of perspectives and subsequently conducted interviews with faculty members and core member alumni.

#### 2.3.1 Novice and senior students

The interviews with novice and senior students were conducted online via the Zoom® platform and consisted of three stages. In stage 1 (lasting approximately 15 min), the researcher shared the purpose of the study, the interview and the rich picture procedures (See Appendix 1 for the rich picture guide). In stage 2, students spent about 45 min drawing a rich picture of a memorable situation they lived that changed their perspective on the medical profession because of the interaction with their peers. Remarkably, most of the novices' drawings were about their experiences in the context of BEM activities. In the third and final stage (lasting 60–90 min), students described the meaning and story behind their drawings without interruption. Next, the interviewer asked about the drawing's elements, including colours, metaphors, use of space and symbols. Finally, the interviewer conducted a semi‐structured interview, with questions informed by the theory of communities of practice^13^ (See Appendix 2 for the interview guides).

#### 2.3.2 Alumni and faculty members

We conducted 45‐ to 60‐min interviews with faculty members and alumni. Interviews with faculty members were held in person, while interviews with alumni were conducted online via Zoom®. Faculty members and alumni did not draw rich pictures, as our focus was on their reflections regarding the impact of student organizations and not on their lived experiences. The interview questions for faculty members and alumni were also informed by the theory of communities of practice.^13^ Additional questions were created for alumni to explore their roles in the BEM and gather their experiences and perspectives about the orientation programme (See Appendix 2 for the interview guides).

Our sample size was guided by the concept of information power, that is, we decided to stop collecting data when the data obtained were rich enough to ground a rigorous understanding of our research question.^22^ All interviews were audiotaped with the participants' permission and transcribed verbatim.

### 2.4 Data analysis

The analysis of our data involved a systematic and iterative process of coding and thematic categorization. To ensure that the analysis was grounded in the participants' actual experiences, we engaged in constant comparison by moving back and forth between the data, codes and themes to develop a comprehensive understanding of the impact of the BEM on students' identity development. Data sources included interview transcripts and rich picture drawings, which were analysed both independently and in relation to one another. A professional translator who spoke the local language translated the transcripts into English. IPKD checked the translated transcripts before sharing them with the research team and provided explanations for specific words that did not have an equivalent in English.

IPKD began the initial, open, line‐by‐line coding of the *original* transcripts of novice and senior students and kept notes from every interview session. Letting the participants elaborate on the meanings, symbols and content of their drawing helped her make sense of the depicted situations. To further familiarize with the data and gain a holistic understanding of the participants' perspectives, we repeatedly reviewed the transcripts and rich pictures. MACF coded the first two *translated* transcripts of novice students. NM, who speaks the local language and has experience in medical education qualitative research, coded half of the *original* transcripts of novice and senior students. NM and IPKD discussed the results in separate meetings. Throughout the coding process, we maintained reflective notes documenting our thoughts, ideas and interpretations. IPKD and MACF weekly discussed the coding and notes, continuously comparing new data and interpretations with previously coded data. Based on the insights gained from previous analyses, we elaborated on and developed the interview guide further. Next, we moved to focused coding, concentrating on deepening and refining the most important and meaningful codes to answer our research question.

A similar process was adopted for analysing the interviews with alumni and faculty members. IPKD coded the *original* transcripts of faculty members and alumni, while NM coded the *original* transcripts of faculty members and reviewed the coding of alumni transcripts. The coding progressed from initial, open coding to focused coding.

Then, the entire research group collaborated to categorize the codes into themes and subthemes. We engaged in preliminary categorization discussions, continuously comparing codes, subthemes and themes from all data (students, alumni and faculty) until reaching a consensus on a final theoretical framework.

We analysed the pictures and interviews in parallel. We explored the pictures in five steps. The first step was to describe only the drawings without making assumptions. The second step was to analyse the use of space, colours, symbols, expressions and metaphors. Then, in the third step, the researchers ‘guessed’ the story behind the picture. All researchers except IPKD actively analysed the picture from step one through three, while IPKD only listened and took notes. In step four, IPKD, who interviewed the participants, shared the story and meaning of the drawn elements. In the fifth step, we gave meaning to the pictures in relation to the research questions. All analyses were audio recorded.

As a final step in analysing the pictures, we organized a 2‐h gallery walk in May 2023. In addition to the authors, five people participated. They were professional medical doctors, nurses and medical students from the University Medical Center Groningen. Some conduct research on PIF and are experienced with the rich pictures technique. The gallery walk consisted of three steps. In the first 10 min, the main author informed the participants about the drawing instructions for the study participants, after which we briefly practiced how to analyse the pictures. Then the participants observed the pictures for 20 min, followed by a 30‐min discussion of their interpretations, including main characters, emotions, use of colours, metaphors and even the stories happening in the pictures. Finally, we spent 40 min categorizing similar pictures and assigning meaning in relation to the research questions. The discussion was audio recorded with participants' permission. The rich picture analysis informed the coding analysis and thematic categorizations.

### 2.5 Research team and reflexivity

This project involved five researchers and an information specialist: IPKD, JF, MACF, JP, NM and TB. IPKD is a PhD student at the University Medical Center Groningen, and this study is a part of her PhD trajectory. She works as a lecturer at the medical school where this research has been conducted for 15 years, since the school was created. She knew all the faculty members who were interviewed and most of the senior students. The higher the seniors' academic year, the closer their interaction with the interviewer, due to the number of meetings in such a collaboration of formal and informal activities. This insider perspective was important to allow the research team to understand the nuances and hidden mechanisms of the socialization process. JF is a professor of Health Psychology interested in the well‐being and personal and professional development of university students. She is familiar with diverse theories on identity development and contributed with her academic perspective on this topic. MACF is a professor in Health Profession Education research with experience in researching the hidden curriculum in medicine and its impact on professional identity development. JP is an andragogue with research experience in the field of burnout, which gave insights into how the mechanisms explored in this study could affect students' engagement with their educational activities. NM is a senior lecturer at the medical school of the University of Alkhairaat, which reinforced the Indonesian perspective. TB is an information specialist with experience in health professions education research. The diverse backgrounds of the team members allowed us to acknowledge our different perspectives and biases and strengthened the rigour of our research and understanding of the participants' experiences and perspectives. During the research process, we constantly reflected on our positions and subjectivities.

## 3 RESULTS

During the data collection and analysis process, we realized that becoming a member of the leading student organization (BEM) played a central role in the socialization process and identity development of novice students.

Students considered it important to become a BEM member because it provided access to exclusive resources and opportunities, such as networking and access to extracurricular activities that could potentially enhance their skills, knowledge and contribute to their personal and professional development. To become a BEM member, all novices had to engage in an orientation programme and join a series of activities aiming for instilling a set of behaviours, attitudes and values professed by this specific academic community. Although the BEM orientation programme was local, the research team—including members from Brazil and The Netherlands with international experience—recognized that the social mechanisms in place might also be present in student organizations in different contexts. We believe that some of the mechanisms we explored may be present to varying degrees in student organizations worldwide.

**FIGURE 1:**
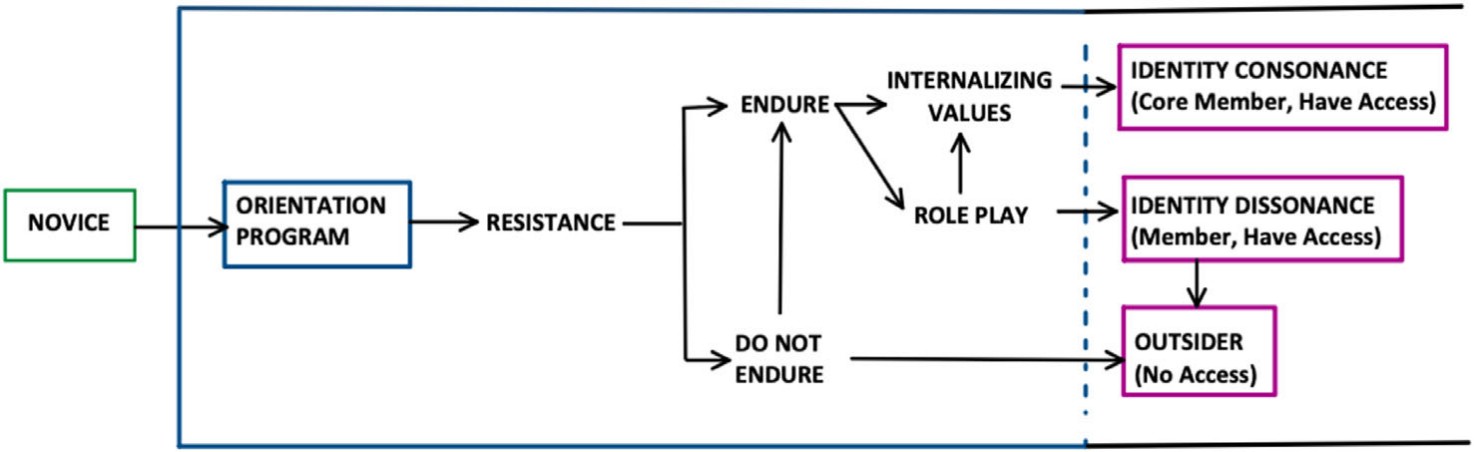
Novice students' responses to the Badan Eksekutif Mahasiswa (BEM) orientation programme. [Color figure can be viewed at wileyonlinelibrary.com]

In the following sections, we will elaborate on novice students' responses to the BEM orientation programme—which ranged from resistance to endurance—and the impact of this programme on students' personal and professional development. We will also reflect on the negotiation process students engaged in, a process that leads to three distinct outcomes: identity consonance, identity dissonance or becoming an outsider (Figure 1).

### 3.1 Orientation programme

The BEM, represented by a team of five second‐ and third‐year senior students, organized the semester‐long orientation programme that ran concurrently with the formal academic programme. The formal curriculum comprised 40 h per week. The orientation programme occurred daily or on alternate days and involved an additional hour before and after the academic schedule on those days, with an extra full day dedicated to the programme on weekends. Attendance at all sessions was mandatory for students aspiring to become members, and failure to comply could result in exclusion. As a novice explained:

‘…usually, [we gathered] every Saturday, we also gathered every morning, every day. It was quite burdensome because, at that time, we had just entered the second block. We were still busy with the initial phase of our studies and needed to adapt. But, early in the morning, we were required to gather, bring food and drinks [for breakfast and lunch], and prepare many things’. (N9)

The orientation programme introduced the medical student community to the novices. Senior students provided training sessions that focused on both academic and non‐academic skills. However, they also discussed the values they believed were central to becoming a doctor. As a novice said:

‘Senior [students] always remind us about the “Triad of Medicine”, which includes hierarchy, camaraderie, and confidentiality’ (N5)

**FIGURE 2:**
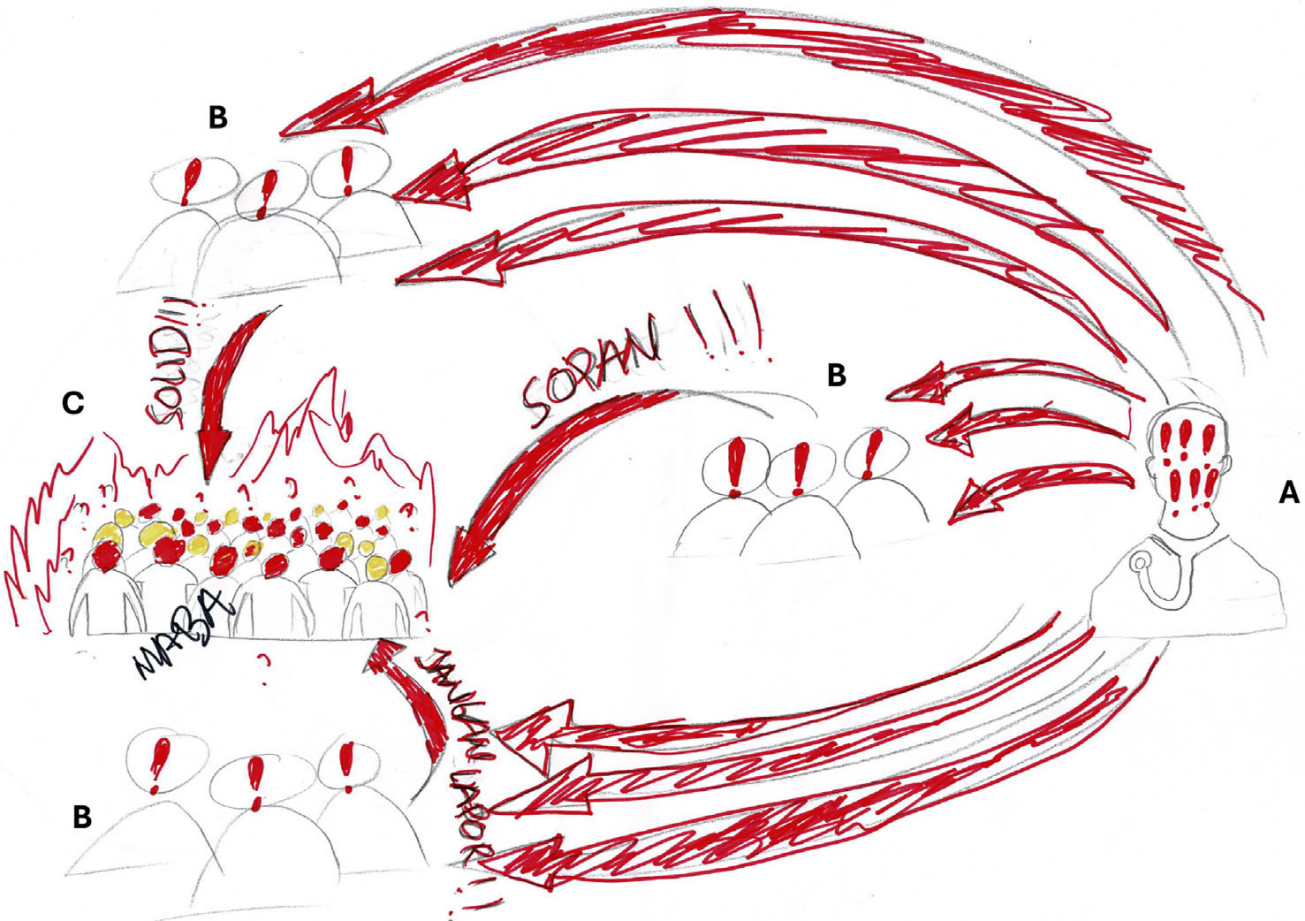
This picture depicts the experience of a novice in the orientation programme. He divided the actors in the picture into three groups: faculty members (a), senior students (b) and novices (c). He explained the hierarchy structure among medical school community members by using big red arrows. The number of exclamation marks inside the heads indicates each individual's level of power. The faculty member has a more vivid figure because he is an expert within the hierarchical system. Possessing more authority, faculty members can easily influence each senior student to obey them, resulting in a power dynamic characterized by dominance and submission. As a corollary, senior students adopt the same attitude and maintain the same behaviours toward the novices as a group. In the drawing, the red arrows represent the forced internalization of BEM values. Senior students consistently reinforce and impart the three values of hierarchy, camaraderie and confidentiality to novices, which is emphasized by the numerous exclamation marks. Consequently, novices often experience feelings of anger (indicated by a red head) and confusion (depicted by a yellow head). (Translation: ‘MABA’ means ‘novices’; ‘solid’ means ‘solidarity’, representing the value of camaraderie; ‘sopan’ means ‘polite’, representing the value of hierarchy; and ‘jangan lapor’ means ‘do not tell anyone’, representing the value of confidentiality). (Picture from N11) [Color figure can be viewed at wileyonlinelibrary.com]

These three values, called the ‘triad of medicine’, represented the implicit norms only documented in the ‘book of cadre’, a guidebook for the orientation programme exclusively accessible to BEM board members. According to senior students, novices had to internalize these values to become part of the medical community. However, as elaborated in the following sections, the way the seniors put these values into practice deviated from their intended purpose, leading to a mismatch that the novices perceived as oppressive (Figure 2).

#### 3.1.1 The first value—Hierarchy: ‘You need to be polite!’

##### The clash between building up respect and trust and forcing obedience

Senior students, who felt responsible for integrating the novices into the community, taught them that hierarchy was the most important value. For the seniors, valuing hierarchy meant showing respect to and obeying senior students and people in positions of power. They believed that being polite was mandatory to respect the hierarchy. For instance, senior students obligated novices to start all conversations with specific sentences to show deference. However, novices, being at the lowest level in the hierarchy ladder, should not expect or receive the same level of respect from faculty members and senior students in return. There was an implicit assumption that by coping with this hierarchical structure, novices would build relationships with senior students that would pay off in the future. As a novice explained:

‘[…] the senior students really emphasize it [that we need to behave politely and respectfully to people higher in hierarchy]. They say, and I also feel it, that in our lives, we'll never know who's going to help us in the future, who we'll deal with, who we'll need help from. When we don't behave politely, and when we don't respect others, we'll end up harming ourselves [our medical trajectory and future careers]. Take, for example, when we novices are not so polite, [then] it's pretty certain the seniors won't feel like helping us much. But when we show them respect, when we […] know how to behave politely toward the seniors and faculty, they'll surely respond warmly to our requests. The seniors will help us out, and if anything happens to us, they'll also be willing to help us.’ (N6)

By fostering hierarchy, senior students gained power and control over the novices. As a result, they expected the novices to obey any commands issued. Disobedience carried consequences, such as being humiliated or physically punished (like having to do push‐ups) in front of the group. A novice said:

‘When I was participating in the BEM orientation program, we could never rest. In the morning, at six or eight o'clock, we had to go to campus. Then … at the campus, the senior [students] yelled at us, punished us, and instructed us to do push‐ups, squats, and jumps. So, we were exhausted when we had to study. Exhausted because the seniors let us down and made us tired [physically and emotionally]. In the evening, we were assigned multiple tasks and stayed up late because of them. This cycle continued the next morning—waking up early and going through it all over again.’ (N2)

These mechanisms of forced obedience were so strong that they might have overshadowed the influence of faculty members and school regulations on novices' identity development. As a faculty member stated:

‘They [novices] are more afraid of their seniors than of us [faculty members], that's evident. Like yesterday. They should have gone home early because they will have an exam today, but [the novices] said “We can't go home early because we have an anatomy support session [an informal class, led by senior students, to provide assistance and support for students studying anatomy],” so they had to wait until late [before they could start studying for the exam]. What I mean is their fear [for not obeying the seniors] […] because, logically, the anatomy support session could be scheduled after the exam, because the exam is more important. So, it's evident that they're more afraid of their seniors than of the school regulations or faculty members’. (FM5)

#### 3.1.2 Second value—Camaraderie: ‘You have to show solidarity!’

##### The clash between nurturing a sense of belonging and suppressing novices' individual voices

To help novices foster a sense of belonging to the medical community, senior students taught them about the value of camaraderie. All novices should recognize, embrace, support, assist and care for one another. They also need to show solidarity and ‘kompak’ (a word in Bahasa Indonesia that means responding with one voice to challenges posed by others). For instance, if a novice made a mistake, the seniors expected all novices to take responsibility and find a solution to their friend's problem together. Seniors called this collaborative process ‘collective responsibility’. To enforce this shared responsibility, senior students adopted a strategy that deviated from their intended purpose. For instance, if one novice misbehaved, the seniors punished the entire group (‘collective punishment’). A senior clarified:

‘[…] We give them [novices] assignments, then we give them … how do we say it … a stimulant to make them understand that if one doesn't complete the assignment, the punishment will be collective. So, for them, it's like their workload increases. This way, they will remind each other to complete the tasks on time.’ (S5)

Seniors also believed that gathering with peers nearly every day could enhance novices` interaction and strengthen their bonds, facilitating a deeper understanding of and respect for one another. Novices had to wear identical clothing to convey the idea that they were equals and possessed equal value, aiming to promote a sense of unity and commonality among them. Seniors expected that cultivating camaraderie among novices would foster a sense of belonging to the medical community. As a senior explained:

‘[…] for instance, helping each other is part of developing fellowship. We don't need to know who we are helping. As colleagues, we should help anyone. Some students choose specific individuals to help, for example, “if they aren't my friends, I won't help.” When we observed this [behaviour], we informed their class leader or approached the newcomers [directly]. We encouraged them to embrace those students [who initially were not their friends]. They [newcomers] may feel like only a few of their friends care about them and others don't. They might feel that way, but their peers actually care about them. These students may feel alone, thinking that only two or three friends are close. Over time, we see them realizing that everyone is a friend. So, when they help, they help everyone, knowing that others will do the same for them’. (S2)

However, when the group decided to support the novice in trouble and respond as a cohesive group, the seniors failed to recognize the group's efforts and solidarity. At the same time, the pressure to react as a group often suppressed students` individual voices. As a corollary, the novices preferred silence over speaking out. Like a novice stated:

‘[…] For instance, if there is a fellow student who asks questions, I actually feel jealous because I want to ask similar questions, but I don't for the good of my classmates. So it is like, we had a two‐hours lecture and then, suddenly, at the end of the lecture, […] the doctor gave us the chance to ask questions. I know how my fellow students feel when someone asks questions just before the lecture finishes. They say, “Don't ask questions” or ask, “Why did you ask questions?” because they want the lecture to finish. I admire the person who has the courage to counter other classmates. When other classmates discourage asking questions just before the end of a lecture, I, who also have questions in mind, feel reluctant to speak out because I am afraid of being blamed by them’. (N4)

#### 3.1.3 Third value—Confidentiality: ‘Don't tell anyone!’

##### The clash between nurturing independence and protecting the organization

The seniors imparted the value of confidentiality by drawing parallels with the responsibilities of doctors entrusted with protecting patients` privacy by keeping their information confidential. However, they expanded the concept of confidentiality by forbidding novices to share what happened during the orientation programme with their relatives, friends outside the medical community and faculty members. In doing so, they intended to encourage novices to solve their problems autonomously without seeking help or advice from their parents, demonstrating maturity as problem‐solvers. Several faculty members supported this ‘call’ for maturity. As a faculty member said:

‘Confidentiality is actually about maturity. Many students tend to report everything to their parents. However, students should handle independently the things that can be resolved by themselves. You just need to share [such issues] with your colleagues; [there's] no need to broadcast them elsewhere, especially when it comes to the educational process’. (FM7)

However, the value of confidentiality was also used to protect senior students from any kind of criticism, potentially creating an abusive and unsafe environment. As a result, novices could not complain about the orientation programme. They felt constrained, isolated and could not express their feelings. As a novice said:

‘They say: “whatever goes on here [in the orientation program] must stay here,” we cannot share it with anyone [except our classmates][…]. We also have this commitment letter stating that we cannot report to faculty members or parents’. (N10)

### 3.2 Oppression

The enforcement of hierarchy, suppression of novices' voices and eventual abusive behaviour of senior students prevented the novices from having autonomy to decide on how to engage with their socialization process. This evolved into a form of oppression, exacerbated by the inherent lack of alignment, and even contradiction, between the values professed by the seniors and the way they behaved toward novices. Moreover, the orientation programme imposed additional stress on novices as they struggled with adapting to the numerous responsibilities during their first year as medical students. Novices felt burdened, humiliated and blocked. A faculty member stated:

‘The things that create confusion are when a newcomer is punished just for mistakenly addressing a senior by the wrong name. To me, that leans toward bullying, especially when it involves physical punishment, like doing push‐ups at seven in the morning and all sorts of things. From my perspective as someone who's never experienced that, it seems unnecessary. Even during exams, if the students can't answer questions, we don't instruct them to do push up either. After all, this isn't the military’. (FM4)

In the following paragraphs, we will elaborate on how novices responded to this oppression.

#### 3.2.1 Awakening to oppression: The spark of resistance

The interviews showed that, at the beginning of BEM's orientation programme, novices tried to resist the oppression imposed by their seniors. They tried to vocalize their concerns and the perceived injustice, while simultaneously defending their positions and integrity. However, the novices` efforts to be heard were met with punitive consequences rather than positive responses from BEM members. This clash contributed to an increase in novices' frustration and anger with the school system. Ultimately, feeling limited in their ability to take action, they developed a sense of helplessness. As an alumnus reflected his experience:

‘[…] As for the system of violence, the reward and punishment system itself, I don't agree. I don't agree. I don't even agree with it because of what I said earlier that this system is old‐fashioned. This system is no longer relevant to … novices, because … it doesn’t make sense. I sometimes … I feel hate, then I don't agree, it's like I want to fight but there's no chance to be able to fight, because if for example we fight we will automatically be expelled from the orientation [program]’. (A3)

After this initial phase of resistance, novices engaged in an internal negotiation process wherein they confronted the decision of whether to persevere with the orientation programme or not. Some students coped with the orientation programme by quickly internalizing the values imparted by seniors. Others experienced identity dissonance, which could culminate in giving up the programme and becoming an outsider. Another group of students adopted a coping mechanism based on ‘role‐playing’—acting as if they had internalized the values while maintaining internal scepticism about the programme. This was a dynamic process. Some students who adopted this ‘role‐playing’ attitude could either abandon the program in the future, become outsiders or eventually internalize the values. This negotiation occurred within the context of persistent pressures imposed by BEM members, leading to frustration, confusion and anger among the novices.

‘I believe the orientation program is merely a formality. It doesn't turn a person into someone kind‐hearted and caring. That's why I also feel, well … somewhat compelled. Because if we neglect helping friends in trouble, we might find ourselves in trouble too. That's why after the orientation program, if someone's in trouble, well…we don’t feel obliged to help them because we won't be punished by senior students anymore’. (N1)

In the following section, we will explore the mechanisms underlying this negotiation process: (1) internalizing the BEM values to secure being accepted as a member of the community, (2) enduring by role‐playing and (3) becoming outsiders.

### 3.3 Outcomes of the negotiation process

#### 3.3.1 Internalizing BEM values and being accepted as a member

**FIGURE 3:**
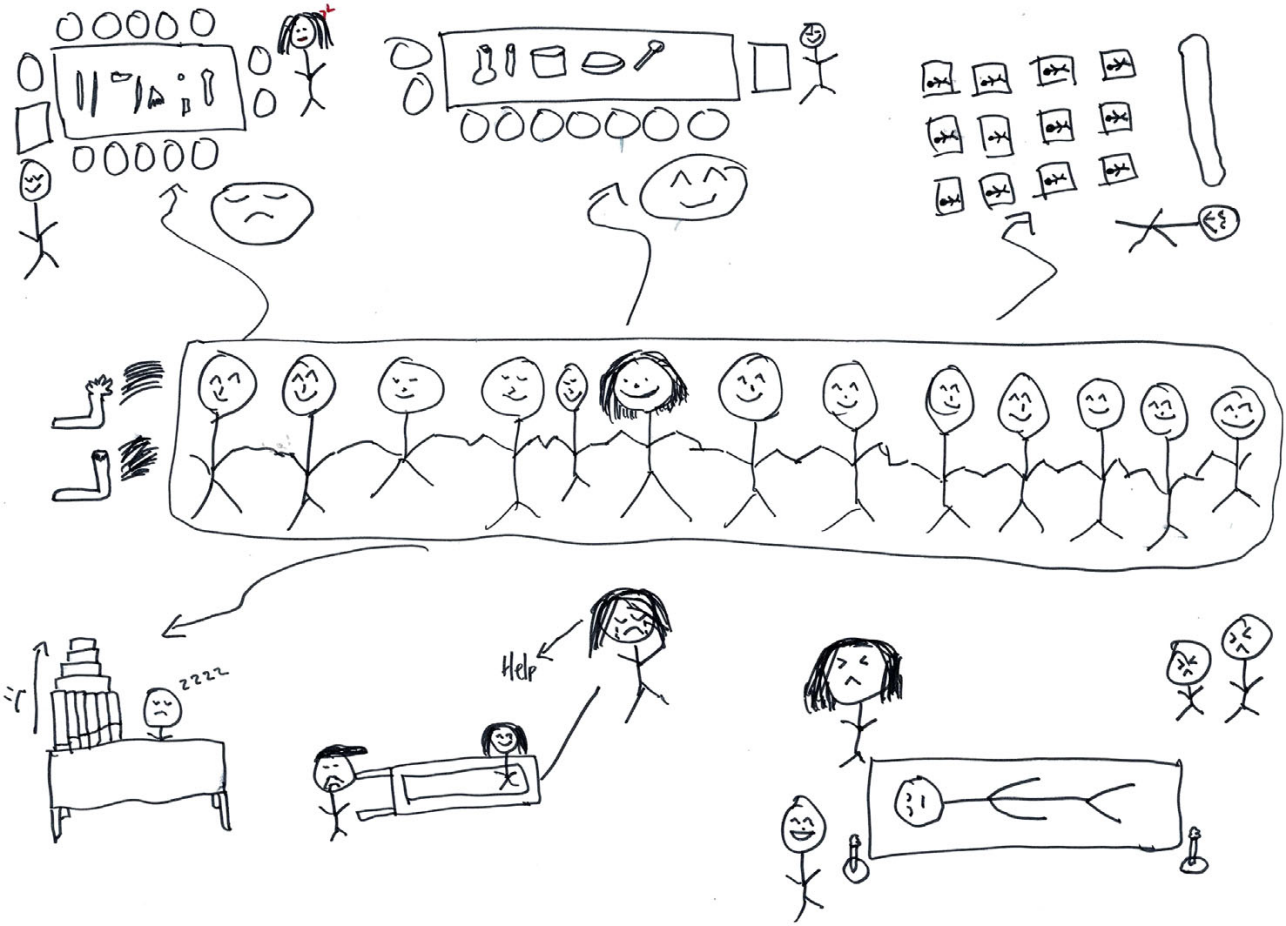
This picture tells a story about a novice who feels content with her peers in a small formal group because she feels connected and supported, as illustrated by smiley stick figures holding hands inside the frame. The stories outside of the frame depict her experiences as a novice, displaying a variety of emotions in each setting. However, such experiences made her stronger, and she learned to handle them in a positive way by making sense of each experience and imagining what she might face in the future when she becomes a medical doctor. The support of her peers helped her cope with difficult situations. (Picture from N6) [Color figure can be viewed at wileyonlinelibrary.com]

Novices who constructed a meaningful understanding of these values were those who perceived the advantages associated with aligning with the community. They believed that early alignment could facilitate their progress throughout their academic trajectory and foster their overall development (Figure 3). Like a novice who felt satisfied with her small formal group explained:

‘So, in the beginning, we may not have been close to each other, each having our own friendship circles. However, as we progressed through the orientation program together, the principle of “one makes mistakes means everybody does” was instilled [in us]. It makes us feel that we have to support, remind, and care for each other, and should not be apathetic. I believe these values are actually not only for orientation purposes; they are also important for me as a human in the future. If I want to become a successful person in the future, I need [to recognize the importance and adhere to] these values. I feel like I am getting the training here…’. (N6)

The decision to internalize the BEM values was reinforced when novices related their experiences lived within the BEM orientation programme to experiences encountered within formal medical training. For instance, when faculty members asserted their power over senior students by questioning and doubting their competencies in front of a group, novices saw their experiences within the BEM programme as a preparation to handle such moments. They recognized that the BEM structure mirrored the overall structure within their formal medical training and reflected the hierarchical nature of medicine.

‘That's why we, as novices, were taught about the triad of medicine. So, when the doctor confronted the senior students, their personalities were scrutinized, all their past faults were revealed: “When your class encountered a problem, where were you? Where was your sense of camaraderie? You didn't help each other.” The main thing is, that whatever the seniors instilled in us, the doctors also applied to the seniors. At that point, I realized that I needed to change my mind from complaining to accepting the situation after witnessing it [in medical practice]. This is why the seniors instilled it in us’. (N11)

This group of students expected to become full BEM members and align with the organization's ideas and activities. They considered their affiliation with BEM pivotal for their overall development. The privileges associated with BEM membership encompassed networking opportunities with senior students and faculty, access to personal and professional training resources and provision of a secure environment conducive to personal and professional growth, collectively referred to as ‘access’. As a senior stated:

‘The first [access] is the sense of brotherhood that we can't find outside the organization. Because we get close to people in the organization … because we engage with them frequently in various activities. The other [access] is … the acquisition of many skills within the organization, which [often] depends on the specialization or identity upheld by the organization’. (S3)

#### 3.3.2 Enduring by role‐playing

**FIGURE 4:**
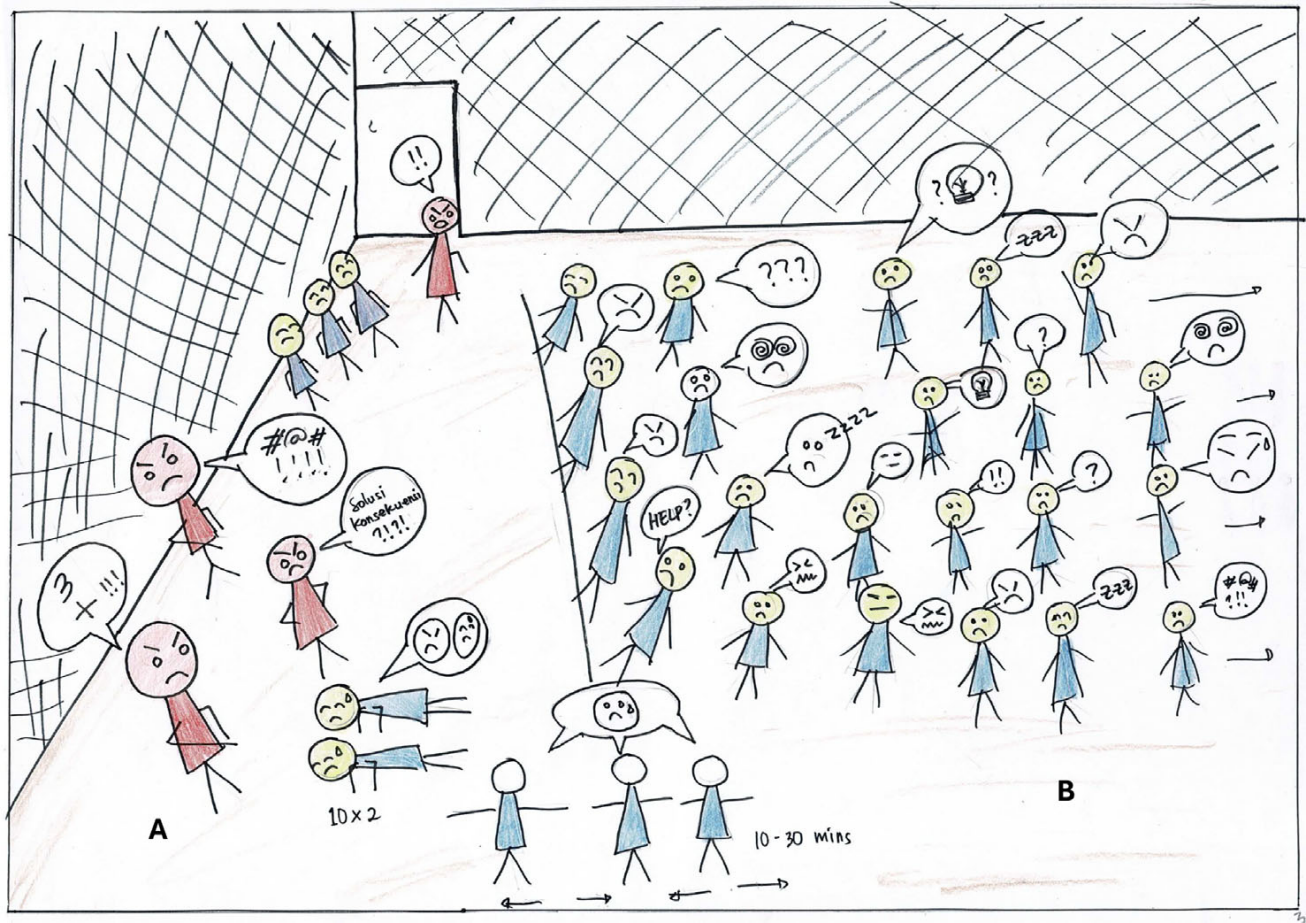
The depicted scene illustrates novices gathered on a futsal field in the faculty. Seniors (a) are depicted larger, expressing anger with red faces and authoritative body language. Most novices (b) are arranged in a straight line, showing varied emotions inconsistent with the thought bubbles. Seniors shout about three values they have taught, demanding solutions with consequences for unsatisfactory responses from novices. Novices' faces are depicted in yellow, indicating tiredness and confusion. In the bottom left part of the picture, various physical punishments are evident. The two class leaders who are doing push‐ups have to take responsibility for their peers' mistakes. (Picture from N9) [Color figure can be viewed at wileyonlinelibrary.com]

A group of novices did not fully internalize the values. Instead, they conformed and adopted a pretence of aligning with the senior students, essentially engaging in a form of role‐playing (Figure 4). There was a contradiction between their intentions and their professed actions, leading to a sense of identity dissonance. Consequently, they grew increasingly frustrated with the oppressive mechanisms at play. As a novice stated:

‘It's just to let other people [senior students] think what they want. We just follow what everyone else does. It's like role‐playing. When a senior asked us to do things during orientation, like push‐ups, we would pretend to be in pain so they would forgive us. It's easy because we can adjust our expressions when the “drama” begins’. (N3)

#### 3.3.3 Becoming outsiders

**FIGURE 5:**
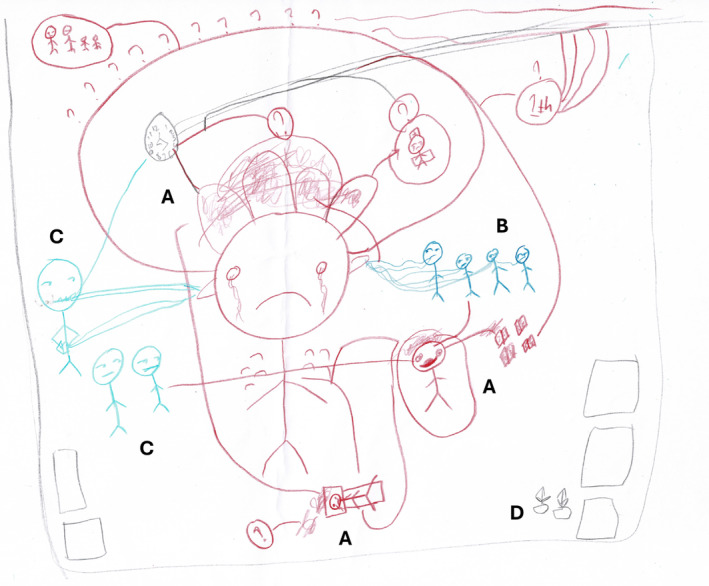
This picture tells the story of a novice (a) who feels isolated by her peers (b). She drew herself in the centre of the drawing as a crying flower, with the revolution taking place in her mind above her head (a). She is cognitively and emotionally overwhelmed, feels isolated and alone on her journey as a medical student and is bombarded by negative comments from both peers (b) and seniors (c) depicted by the green lines. Some seniors try to give advice and positive affirmations, as shown by the red line. However, she feels overwhelmed by it and can only show a depressive and confused smile. She needs help but does not know who will understand her situation. She drew grey flowers (d) at the bottom right of the picture, symbolizing her current sad condition. She embraced the metaphor of a flower, finding strength in its ability to weather storms and thrives the next season. Beyond their outward beauty, flowers serve as vital contributors to oxygen production, reflecting the multifaceted growth she envisioned for herself. (Picture from N12) [Color figure can be viewed at wileyonlinelibrary.com]

Some novices ended up as outsiders, which was a result of a complex interplay of external and internal factors (Figure 5). External factors, such as parental disapproval of the orientation programme's methodology and its substantial time commitment, which their parents perceived as irrelevant to the trajectory of becoming a doctor, played a pivotal role in the novices' struggle. Despite their desire to participate in and endure the orientation programme, they struggled with resisting their parents' wishes, ultimately leading to their decision to withdraw from the programme. Additionally, internal factors, such as physical illness, could result in novices being dismissed due to mandatory attendance requirement. Furthermore, some novices became critical and dissatisfied with the programme, perceiving it as not meeting their expectations and failing to address their needs. The programme neither aligned with their initial expectations nor justified the sacrifices they had to make.

Being an outsider in the community could pose challenges, especially in the first year. Outsiders frequently engaged in different informal campus activities than most of their peers, leading to feelings of discrimination and isolation. As a novice who felt isolated and confused stated:

‘… They [members] also have a [WhatsApp] group that didn't include me, so they have a separate group, which I find very confusing. Why did they create a group without me?’ […] My friends, maybe they are avoiding me. [For example,] one day, in preparation for an exam, they might have been taught by senior students [as part of the introduction program]. When they come together and discuss [the exam], they are open and communicative, but when I join them, they suddenly start signalling each other to keep quiet and not share the content, which leaves me feeling sad, even though I know this is the consequence of being an outsider. (N12)

## 4 DISCUSSION

Through a detailed exploration of a specific student organization, we gained valuable insights into the broader influence of student organizations on socialization processes in the context of the PIF of medical students. First, we noticed that student organizations may instil values and promote behaviours outside the formal curriculum radar. Second, we found that centralized student organizations could use their power to control access to both personal and professional development opportunities, such as extracurricular training and networking. Third, we saw how a mismatch between students' personal and professional values and the values professed by representatives of the student organization can lead to a sense of identity dissonance, intensified by an often hierarchical power dynamic. In this complex interplay, students who internalize the organization's values find a space for growth. However, students who do not align with these values experience identity dissonance and oppression, which undermines their socialization processes in a vicious cycle of exclusion.

While our study was conducted in a single institution, the insights provide valuable perspectives on the functioning of student organizations and the mismatch between senior students' intentions and actions. Even with good intentions, senior students engage in oppressive actions that can harm newcomers. This condition suggests a hazing culture that has been perpetuated across generations of students. Our findings resonate with those of Owen et al.,^23^ who confirmed the prevalence of hazing across various student organizations, particularly in fraternities. The medical education community, including students, typically holds a negative view on hazing and demands for interventions to mitigate such practices; however, these hazing practices still exist in different parts of the world. For instance, in Thai universities, a hazing tradition known as ‘rap nong’ or ‘welcoming freshmen’ involves senior students coercing and pressuring freshmen into performing absurd acts to demonstrate submission and unity, perpetuating a culture of hierarchy and violence.^24^ Similarly, initiation rituals with extreme hazing practices for newcomers in the Netherlands received negative headlines in national news. A Dutch independent university newspaper posits that these rituals are deeply rooted in tradition and function as a mechanism for selecting motivated members and fostering unity.^25^ In a study from Brazil, hazing practices were related to decreased well‐being of medical students.^26^ We hope our study will function as a call for medical schools around the globe to pay closer attention to the socialization mechanisms in place within their respective student organizations and to explore how these organizations impact students' PIF.

In the context of this study, the student organization and its members were empowered by the hierarchical structure within the local medical community. Senior student members of the organization believed in the goodness of the values they imparted, feeling a strong sense of responsibility to help novices adapt to their new environment. Having observed their teachers' behaviours within the hierarchical system, they openly replicated these actions when interacting with novices. However, despite their good intentions, they remained unaware of the mismatch between their intentions and actions, such as the mismatch between building up respect and trust versus enforcing obedience. This lack of awareness led to feelings of oppression among novices, highlighting the importance of aligning intentions with actions to foster a positive learning environment.

Addressing this mismatch is crucial for mitigating the oppressive dynamics experienced by novice students. Students need to be aware of and evaluate whether the values they promote align with those expected within the medical profession. While the BEM's values are derived from those of Indonesian physicians, the ways in which they are implemented in practice certainly differ. This discrepancy arises as students interpret, adapt and enact professional values within the context of the medical student community, often without reflecting on the practical consequences of their actions.

Practical wisdom is a philosophical concept proposed by Aristotle and referred to as ‘phronesis’.^27^ It represents ‘a fundamentally practical form of reasoning concerning human action—an intellectual virtue that enables us to judge what we should do in a given situation. It is a multifaceted concept encompassing reasoning, action, context, and appropriateness’.^27^ Practical wisdom represents the integration of knowledge and competence with appropriate experience, judgement and situational understanding.^27^ The senior students lacked this wisdom and were unable to reflect upon the consequences of their actions, leading them to perpetuate the existing oppressive system without critical reflection or evaluation. We believe that by promoting critical reflection and engaging in a democratic dialogue with their peers and teachers, students could not only develop practical wisdom but also challenge and contribute to the reform of oppressive structures within medical schools.^28^

We observed how a group of novices, in their negotiation process of acquiring a new identity, experienced repression or depersonalization. Repression is a defence mechanism that happens unconsciously, followed by reduced memory for unpleasant or forbidden thoughts, effects and wishes.^29^ Novice students experienced repression as they felt compelled to conform to the rules, norms and values of the student organization. In alignment with our findings, Rabow et al.^30^ observed how novices repressed their personal values to fit in the community and expressed a fear of being humiliated by senior students. Violato et al.^31^ observed a tendency among novices to obey their seniors and avoid conflicts to maintain harmonious interactions. Repression prevents medical students from engaging in meaningful reflections about their identity development, which hampers their sense of autonomy and capacity to enact change.

Repression can also be accompanied by depersonalization, which is characterized by a diminished emphasis on personal distinctions due to a lack of distinctive cues and leads to increased similarity within a group.^32^ Depersonalization may happen in the medical community, because there is a tendency toward homogenization, neglecting students' personal aspects such as previous social identities and personality traits. Students often conform to this homogenization because they are trying to acquire a collective identity shared by their peers and prove that they can fit into their new role and enjoy membership.^31^, ^33^

Repression and depersonalization are detrimental as they have the potential to induce identity dissonance. As highlighted by Sternszus et al.,^34^ repression and depersonalization can be seen as forms of identity threats that arise because medical school, as a community, tends to favour norms and values of the culturally dominant group, emphasizing socialization as the experience of assimilation toward culturally dominant identities. However, the identities and beliefs of this dominant group do not necessarily align with the professional identities and professional norms we aim to promote as medical professionals and educators. Consequently, when novices fail to adhere to this dominant identity, the identity dissonance they experience can manifest as feelings of anxiety, frustration and inadequacy, prompting individuals to adopt maladaptive coping mechanisms. Ultimately, these experiences may result in adverse outcomes such as academic attrition or marginalization,^6^, ^35^, ^36^ a low level of psychological well‐being^37^ and burnout.^38^

Students who successfully internalized the values of the student organization became core members, experiencing a profound sense of belonging that reciprocally sustained them. After having experienced the privilege of becoming core members, the insiders ended up justifying the organizational culture. However, they may have been susceptible to ‘survivorship bias’, tending to focus on the benefits and successes of those who thrived within the system and overlook the experiences of those who encountered failures or dropped out. Such a narrow perspective may have skewed their understanding of the true challenges and limitations of the educational environment, hindering their ability to critically assess its effectiveness and potential shortcomings. Consequently, the senior students perpetuated the often oppressive existing system. To change the tide and challenge the system, students must engage in critical reflection.

### 4.1 Practical implications

The impact of a student organization on identity formation is complex and context‐dependent. This context encompasses not only the diverse nature of student organizations but also the unique characteristics of individual students, the overarching culture within the medical school and the broader cultural context of the country. We believe it is crucial to refrain from adopting a prescriptive stance, classifying these organizations as inherently good or bad, as such labels oversimplify their multifaceted influence. To understand this multifaceted influence, novices, seniors and faculty must reflect critically on the behaviours and values that constitute the institutional culture to avoid reproducing systems of oppression.

Integrating critical pedagogy, as advocated by Paulo Freire,^28^ can be a valuable framework for reflecting on such a culture. By embracing critical pedagogy, teachers can foster dialogue beyond mere instruction, engaging in a collaborative exploration of PIF within the context of student organizations. This approach encourages an environment of open and honest exchange of ideas, creating space for mutual learning and understanding. Faculty development initiatives should prioritize reflective practices, enabling teachers to continually assess and adapt their approaches in response to the evolving dynamics of student organizations.

Embracing critical pedagogy requires teachers to cultivate epistemological curiosity as a foundational mindset. Epistemological curiosity, first coined by Berlyne^39^ and adopted by Freire,^40^ encompasses an inner drive to challenge assumptions, including self‐criticism, and engages in critical inquiry to make sense of oneself and others within the world. It involves nurturing a shared interest in critical thinking, questioning and mutual knowledge exploration in a context of openness to the other. This openness creates a feeling of unfinishedness, which is vital to broadening previous understandings and renewing the culture.^40^

Epistemological curiosity also demands alignment between reflection and action by adopting a praxis in which behaviours are faithful to the values that motivate them.^40^ However, if teachers do not adopt the same critical stance, they want to cultivate in students, such practices lose their impact. Teachers need to show the same fidelity to critical reflection they want students to develop—critical reflection must be modelled. Therefore, teachers need to be brave to expose their vulnerabilities and blind spots, opening themselves to criticism and feedback, and taking action to improve the educational system.

After acquiring proficiency in implementing critical reflection and comprehending the complexities within the student organization, teachers can collaborate with students to co‐construct programmes that support identity formation for novices. Effectively cultivating such programmes requires collaborative efforts between teachers and student organizations, emphasizing the need to adjust power dynamics within the hierarchical structures in the medical education community. This approach seeks to establish a co‐constructed process between faculty and student organizations, deliberately departing from the conventional ‘power over’ model to embrace a more egalitarian ‘power with’ paradigm.^41^ This intentional shift ensures that the programme reflects a diversity of perspectives, acting as a catalyst for transformative change in entrenched power dynamics.^41^ Embracing the ‘power with’ paradigm entails active contributions from both faculty and students, transcending traditional hierarchical boundaries and fostering a more inclusive and participatory educational environment.

### 4.2 Strengths and limitations

Our study showcases the power of one student organization in reproducing implicit cultural norms that shape students` personal and professional development. Although we believe that we observed a social process common to student organizations in general, we need to be careful when generalizing such findings. The cultural context where this study was performed is marked by a collective perspective, which means that becoming part of the group is highly valued, and ending up as an outsider has an intense and negative psychological impact. It is possible that in individualist cultures, this impact may be minimized. We also need to be mindful that the same organization that reproduces certain systems of oppression is also very effective in transferring knowledge and skills to novices and in creating social bonds and supportive social systems. This ambiguity adds complexity to understanding the dynamics of such social interactions. The research group, connected to and mobilized by the oppression lived by the novices, may have overlooked the positive influences of the organization.

## 5 CONCLUSION

Our exploration into the dynamics of a student organization has illuminated the significant impact they wield on the formation of novices' professional identities. The realization that cultural aspects, encompassing values and norms within these organizations, play a pivotal role in shaping this identity underscores the urgent need for transformative change. This change, however, is not a solitary endeavour. It demands a team effort from faculty, teachers, student organizations and novices.

## AUTHOR CONTRIBUTIONS

**Indah Puspasari Kiay Demak:** Conceptualization; writing—original draft; writing—review and editing; methodology; investigation; formal analysis. **Jelle Prins:** Writing—review and editing; methodology; supervision; conceptualization; formal analysis. **Nur Meity:** Writing—review and editing; formal analysis. **Tineke Bouwkamp‐Timmer:** Writing—review and editing. **Joke Fleer:** Supervision; writing—review and editing; conceptualization; formal analysis; methodology. **Marco Antonio de Carvalho‐Filho:** Conceptualization; formal analysis; methodology; supervision; writing—review and editing.

## CONFLICT OF INTEREST STATEMENT

The authors declare that there is no conflict of interest.

## ETHICS STATEMENTS

Ethical approval was obtained from The Medical and Health Research Ethics Committee of the Faculty of Medicine University of Tadulako in May 2022 (3346/UN28.1.30/KL/2022).

## Supporting information


**Appendix 1.** Drawing Technique Guide “Rich Pictures”.
**Appendix 2.** Interview Guides.

## Data Availability

The data that support the findings of this study are available from the corresponding author upon reasonable request.
