# Supplementary material for: Under the radar: How participating in a student organization can shape medical students' professional identity
Source: Med Educ. 2025 Feb 10;59(7):704–18. doi: 10.1111/medu.15609 (PMC12198712; doi:10.1111/medu.15609)
Supplement: Supplementary file 1 — Appendix 1. Drawing Technique Guide “Rich Pictures”. Appendix 2. Interview Guides. [file MEDU-59-704-s001.docx]

**Appendix 1**

**Drawing Technique Guide "Rich Pictures"**

1. The interviewer starts by describing the steps of the interview. Students will draw one picture of their relationship or interaction with peers (the same class or seniors) related to professional identity formation.
2. **Professional identity** is “*a representation of self, achieved in stages over time during which the characteristics, values, and norms of the medical profession are internalized, resulting in an individual thinking, acting and feeling like a physician*”
3. The researcher is interested in exploring the nature of students' relationships with their peers. What types of relationships do they have, and how do these relationships influence their perceptions of the medical profession?
4. The pictures that students will draw are rich pictures. A rich picture is a visual representation of a particular situation intended to show the experience in all its complexity. It aims to capture the context and its elements, the people, their connections, relations, and emotions. Also, include the perspective and emotional aspects of people involved in drawing. These different perspectives all add to the richness of the situation.
5. Things to consider when drawing:
6. use as few words as possible
7. not make the drawing like a cartoon (do not structure the picture, just draw one picture. Otherwise, we might lose the complexity of the experience).
8. Students can use both coloured pencils and markers or just one of them. Be free to draw the picture. And no need to worry about the pictures. It does not have to be artistic or understandable. Because you will share the story of your picture in the next stage with the interviewer.
9. These are examples of Rich Pictures:

**Figure 1 (van Duin et al, 2021)**


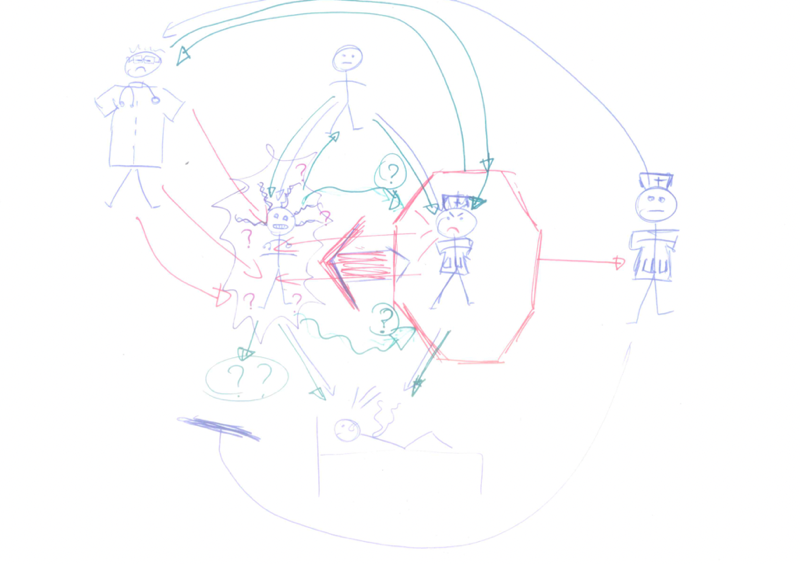


**Figures 2 and 3 (Ribeiro et.al, 2020)**


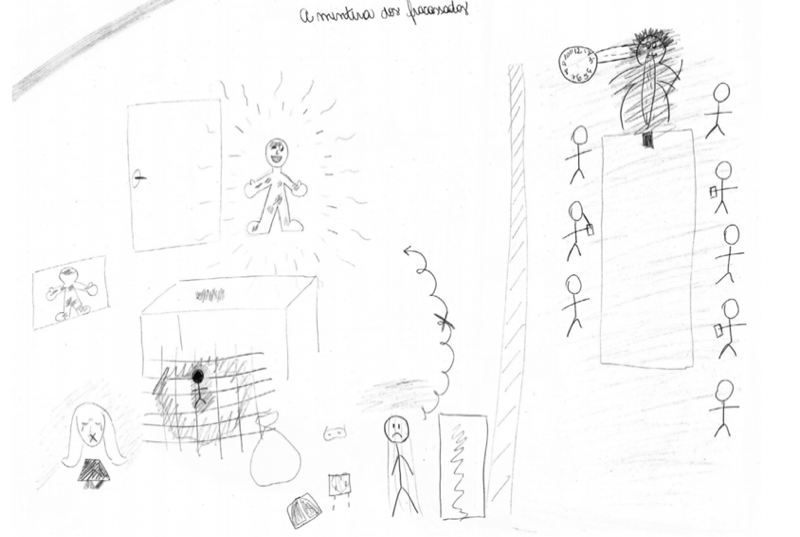


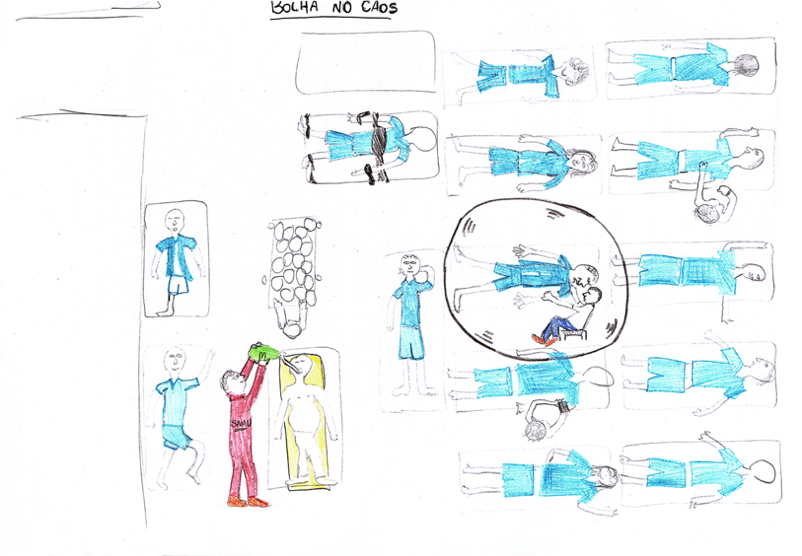


1. Students will draw for a maximum of 40 minutes. The interviewer and students will leave the meeting during the drawing. While drawing, students can drink or chew candy. After they finish the drawing, students will send a notification and pictures via WhatsApp. Then we will rejoin the online meeting.
2. Next, the interviewer will request the student to tell the story behind the picture student in detail and explain all aspects drawn in the picture. Students should feel relaxed as much as possible and do not have to feel rushed when sharing the stories. After that, we will look at the picture together. The interviewer will ask students to explain what they have drawn and ask further questions to help me understand the situation. After that, we will talk about your experiences. This part will take approximately 30-60 minutes.
3. All stages will be audio-recorded in accordance with permission from the students.
4. After the interviewer explain all the above steps, students can ask questions regarding the procedures, the rich picture drawing, or professional identity.
5. Finally, the interviewer invites students to draw a lived experience (**the most memorable experience)** when they interacted with peers that changed the way they see themself and the medical profession.

**Appendix 2**

**Interview Guides**

**Novice students**

- 1. How is it for you to be a medical student? (removed)
  2. How is your relationship with your peers (same level and seniors)?
  3. How is your relationship with the student organizations? (removed)
  4. How is your relationship with the community of students?
  5. How is your relationship with the faculty? (removed)
  6. How is your opinion about the BEM orientation programme? (added)

**Seniors students**

1. How is your relationship with the novice students?
2. How is your relationship with the community of students?
3. How is your relationship with the student organizations?
4. How is your opinion about the BEM orientation program?

**Faculty members**

1. How do you think about student relationships related to their professional identity?
2. How do you think about student organizations related to the professional identity development of undergraduate students?
3. How do you think about your roles in the student organizations?
4. How do you think about the BEM orientation program?

**Alumni**

1. How is your relationship with BEM?
2. How do you think about the BEM orientation program?
3. How was your experience with the BEM orientation program?
